# Memoirs of the Imperial and Royal Academy of Sciences and Belles Lettres of Brussels

**Published:** 1782

**Authors:** 


II. *Memoires de L'Academie Imperiale et Royale des Sciences et Belles Lettres de Bruxelles,* i. e. *Memoirs of the Imperial and Royal Academy of Sciences and Belles Lettres of Brussels,* 4to. Brussels, Vol. I. 1778. Vols. II. and III. 1780.

THE Academy of sciences at Brussels, founded in the year 1769, have already published three three volumes of their tranſactions. Each volume is divided into two parts, one of which under the head of *Journal des Seances*, exhibits a view of the buſineſs of each meeting, together with an analyſis of ſuch papers as have been read before the academy, and which they have not thought fit to publiſh at length. The ſecond part of the volume is allotted to the Memoirs.

The FIRST of theſe three volumes affords us only two medical papers. One of theſe is *an account of the mineral waters of Sauchoir*, by the Abbè DE WITRY; the other is written by M. DE BEUNIE, phyſician at Antwerp, and contains *ſome obſervations on the diſorder, which is ſometimes occaſioned by the eating of muſcles.* The author aſcribes it to a ſmall ſea inſect, a ſpecies of *ſtella marina*, which ſometimes lodges itſelf in the muſcle, and whoſe ſpawn is ſo cauſtic, that even when applied outwardly to the ſkin, it produces itching and ſwellings that are in a high degree painful. The remedy preſcribed in theſe caſes by Dr. de Beunie is vinegar; and he recommends abſtinence from theſe ſhell-fiſh during May, June, July, and Auguſt, the months in which the inſect in queſtion is generally to be met with.

We proceed now to the SECOND volume—

The

The firſt part or *Journal* contains an analyſis of a *Memoir on the contagious diſeaſe of horned cattle*, by the Abbè NEEDHAM, director of the academy. This author divides the diſeaſes of organized bodies into two claſſes, the firſt of which includes ſuch as are inflammatory from an exceſs of vital power, and the ſecond thoſe which are putrid or gangrenous from a defect of that power. He ranks the contagious diſeaſe, which is the ſubject of his paper, among the latter, and in the treatment of it recommends antiſeptics, and particularly common ſalt, as the moſt efficacious remedies.

In the ſecond part of the volume we find *a Memoir on the pernicious effects of muſcles*, by M. DU RONDEAU, which is intended as a ſupplement to Dr. de Beunie's paper on the ſame ſubject juſt now ſpoken of.

Three caſes are related in the preſent Eſſay, which prove that muſcles are as liable to produce dangerous effects in April, September, and October, as in either of the four months mentioned by M. de Beunie. M. du Rondeau is convinced that the boiling of muſcles does not deprive them of their deleterious qualities. This fact, we believe, has long been pretty generally known. He next preſents us with a view of of the ſymptoms produced by the poiſon of theſe ſhell-fiſh, and of which, as it is related with great accuracy, we ſhall give a literal tranſlation.

"The ſigns which announce the noxious ef-

" fects of boiled muſcles, are an univerſal unea-

" ſineſs or numbneſs, that commonly takes place

" three or four hours after they have been eaten.

" Theſe ſymptoms are ſucceeded by a tightneſs

" of the throat, a ſenſe of heat about the head

" and eyes, immoderate thirſt, nauſea and ſome-

" times vomiting. If the patient has the good

" fortune to vomit up the whole of the veno-

" mous matter, this evacuation is generally ſuf-

" ficient to ſtop the progreſs of the complaint,

" but if he brings up none or only a part of the

" noxious ſubſtance, the diſorder becomes more

" or leſs alarming, according to the quantity of

" deleterious matter in the firſt paſſages, and

" according to the particular conſtitution of the

" patient. The want of a ſufficient evacuation

" by vomit encreaſes the tightneſs of the throat,

" and the ſwelling of the face, eyes, and tongue;

" all the parts within the mouth appear inflamed,

" and as it were excoriated, and the redneſs ſoon

" ſpreads to the outer ſurface, appearing firſt in

" the face, and extending from thence to the

" neck,

" neck, breaſt, and abdomen, and by degrees
" over the whole body.

" This particular eruption is the ſymptom the " moſt diſtinguiſhing and characteriſtic of the " malignancy of muſcles; it is conſtantly ac- " companied with a kind of delirium, with a " ſingular uneaſineſs, and an inſupportable itch- " ing; it has no affinity with the eruption pro- " duced by the eryſipelatous fever, with the " ſcarlatina, meaſles, *purpura urticata*, or any " other known ſpecies of red eruption: it has " theſe particularities, viz. that it never appears " unleſs muſcles have been eaten; that it is not " preceded by fever; that it is accompanied by " ſymptoms which appear united in no other " diſeaſe; and laſtly, that the whole ſurface of " the body, tho' redder than in any other eruptive " diſeaſe, appears as it were ſpotted with an in- " finite number of red ſpots of a deeper red than " the reſt of the ſkin. Theſe points are infi- " nitely ſmaller than a millet ſeed; if we exa- " mine them through a lens, we ſee diſtinctly " that they are the openings or pores of the " cuticle, which leaves minute ſpots of the cutis " expoſed to our view, while the redneſs which " is ſeen only through the epidermis, appears of " a paler hue."

Our author, as we have feen, fuppofes this eruption to be an effect peculiar to mufcles, but in perfons of a particular idiofyncrafy we have obferved it brought on by falmon, Peruvian bark, and other fubftances.

The hiftory we have tranfcribed is followed by an account of two anomalous cafes; in one of which the patient was afflicted with violent fpafms, without any attendant erruption. No medicines could be adminiftered by the mouth, but relief was obtained by glyfters. In the other the difeafe terminated in a gangrenous fore throat, from which the patient with difficulty recovered.

M. du Rondeau agrees with M. de Beunie with regard to the efficacy of vinegar given internally as an antidote to this poifon. He has likewife experienced excellent effects from its external ufe. He affures us that by wafhing the whole furface of the body for about half an hour, with vinegar and water, he has feldom failed to remove the difagreeable itching that accompanies the eruption, and to bring on a fweat which has fpeedily terminated the complaint.

The paper concludes with an account of a pretty certain method of depriving mufcles of their poifonous quality. It confifts in wafhing them firſt with water and afterwards with vinegar previouſly to their being boiled in an earthen pot with vinegar and water, and a few grains of Jamaica pepper. Similar precautions are to be taken if the fiſh is dreſſed in its ſhell. This practice, we are told, has been adopted by upwards of an hundred families, and with the moſt uniform ſucceſs.

At the end of the volume are extracts from two meteorological diaries kept at Bruſſells in 1775 and 1776.

The THIRD Volume contains *an account of the Medicinal Leech*, by M. DU RONDEAU, accompanied with an engraving. This inſect, which our author informs us is not deſcribed by Linnæus, or any other writer, is compoſed of 105 cartilaginous rings covered by the cutis, and connected by ſtrong thick muſcles that are endued with ſingular irritability. Its mouth is oval (not triangular, as ſome writers aſſert) when it fixes itſelf to any ſubſtance, and quadrangular when the animal is at reſt. The teeth, which are three in number, are rounded, white, cartilaginous, moveable at their baſis by means of a ligament, and ſo ſituated that they ſerve to pinch the ſkin while it is drawn upwards by the fuction of the mouth. Contiguous to each tooth is a fort of minute nipple, for fo our author terms it, compofed of an elaftic fpongy fubftance, and terminating in a membranous canal furnifhed with a valve. This canal leads to the ftomach, which is a large mufcular bag, communicating with the inteftinal canal. The latter, begins about the 22d or 23d ring, and extends along the two fides of the infect to the anus, forming twelve bags on each fide, and is furnifhed with an infinite number of valves which prevent the return of the food towards the ftomach.

This infect is an hermaphrodite. Its uterus, which is fhaped like a pear, is fituated immediately under the ftomach, and the neck of it terminates in a membranous canal, which opens externally between the 24th and 25th ring. A feminal duct from each of the two *vesiculæ feminales* likewife opens into the fame canal, the mouth of which is furnifhed with a ring formed by the fkin. A little under this ring is fituated the heart, which is a flefhy bag of a conical but irregular fhape, and furnifhed at its bafis with an appendage which feems intended for an auricle. The heart is attached to the back by means of the large veffels.

Our author has not been able to difcover any organs of vifion or hearing in this infect. It is likewife deftitute of lungs; and he has found by experiments that its life can be fupported in oil, or under an exhaufted receiver, as well as in water or the open air. He has obferved, however, that although it is capable of living for a long time without air and without food, yet that it cannot take the latter *in vacuo*, for on placing four leeches and fome frefh blood under the receiver, they immediately began to fuck the blood, but quitted it as foon as the air was exhaufted. This experiment was repeated feveral times.

This volume likewife contains; 1. *an Effay on the poifonous quality of lead, by* M. de Beunie, which though judicioufly written affords us no new obfervations on the fubject; 2. *an Effay, by* Abbè Marci, *on the beft means of tinning veffels for culinary ufes*, in which he recommends block-tin for this purpofe; 3. *an hiftorical and phyfical effay on the* orichalcum *of the ancients; preceded by fome obfervations on the* lapis ærofus *of Pliny*, by M. de Launay. The author's views in this paper are to prove that the orichalcum was not, as fome have fuppofed, a metal which is no longer known to us, but a compofition of copper and zinc, fimilar to our brafs and

Pinch-

Pinchbeck; and that Pliny has fallen into an error in giving the name of *lapis ærosus* to lapis calaminaris upon a supposition of its being a copper ore. Much erudition is displayed in these enquiries. 4. *Results of meteorological observations made in the year* 1770 *at Franeker in Friesland,* by M. Van Swinden, *professor of philosophy,* &c. 5. *Extracts from three meteorological diaries kept at Brussels in* 1777, 1778, and 1779.

